# Triple Assessment in Diagnosis of Thyroid Nodules and Its Comparison With Histopathology

**DOI:** 10.7759/cureus.36021

**Published:** 2023-03-11

**Authors:** Elakkiya R, Sivamarieswaran R, Athira Gopinathan, Balamurugan R

**Affiliations:** 1 General Surgery, SRM Medical College Hospital and Research Centre, Chennai, IND

**Keywords:** diagnosis, malignancy, clinical examination, fine -needle aspiration, ultrasonography, thyroid nodule

## Abstract

Background: Thyroid nodules are more common than previously realised, and the rate of prevalence is hugely impacted by the method of detection and their easy access. No single test is sufficient to access the thyroid nodule at any given time. Hence this necessitates the need for clinicians to use an evidence-based protocol for their assessment and diagnosis.

Aims and objective: To determine the likelihood of malignancy in individuals who have thyroid nodules of any size, by a) performing a triple assessment, including a history and physical examination, an ultrasound of the neck and fine needle aspiration and cytology (FNAC) b) predicting the percentage of correlation between findings of malignancy on FNAC and final histopathological diagnosis c) identifying and validate individual risk factors in the clinical examination and ultrasound imaging that point towards a nodule being malignant

Methods: Patients presenting with thyroid nodules in a clinically euthyroid state were studied over a time period of 18 months. Seventy-five patients were included in this study. Patients having external cytology and ultrasonography reports were reassessed if they consented to the study. If the pathologists thought the smears were sufficient, slide reviews were accepted. A senior consultant conducted the clinical evaluation. Prior to doing the FNACs, the designated radiologist performed the majority of the ultrasonograms. If the physicians believed it was necessary, ultrasound-guided FNACs were performed. According to Bethesda criteria, the cytology was reported. The outcome of the histopathological analysis was used as the gold standard for diagnosis in this investigation.

Result: Out of 75 patients included in the study, the older age group (50-70) patients had mostly malignant lesions (92%). In the younger age group (20-39), about 77% had benign lesions. Benign lesions were more common in females than males according to the histopathology study. Seventy-three percent of fixed swellings turned out to be malignant. About 86% of patients who had extrathyroidal extension ended up being found to have malignant lesions but even 41% of patients who didn’t have any extrathyroidal extension also turned out to be having malignant lesions. However, the presence of pressure symptoms didn’t necessarily translate to being an indicator of malignancy. Ninety-seven percent of patients who had punctate microcalcifications turned out to have malignant lesions. Hypoechogenicity on imaging also is an important marker of malignancy, with about 87% of patients who had hypoechogenicity having malignant lesions proven on histopathology. All the patients who had solid lesions on imaging were proven to have malignant lesions. About 77% of patients who had cystic features ended up having benign lesions. Hence, it is a very significant marker. Intranodular vascularity, taller than wider lesions and positive lymph nodes on imaging were proven to have malignant lesions. FNAC is an important diagnostic tool. It is made out that the reporting of FNAC more or less matched the histopathological diagnosis in almost all categories.

Conclusion: There are definite correlations in the role of triple assessment as a standard protocol in the diagnosis of thyroid nodules and guiding its management.

## Introduction

Thyroid nodules are more common than previously realized as the rate of prevalence is hugely impacted by the method of detection [1]. A mere clinical examination yields a prevalence between 4% and 7% [2,3], but the use of newer imaging techniques like high-resolution ultra-sonogram yields a prevalence anywhere between 20% up to 76% among adult subjects. Numerous nonpalpable nodules, or incidentalomas, in the thyroid can also be found now using these highly sensitive imaging tools. During autopsy studies the prevalence of thyroid incidentalomas ranges from 30% to 60% whereas clinically palpable thyroid gland on imaging shows a prevalence of 13% to 50% [4].

The foremost issue which makes a prompt and accurate diagnosis of the thyroid nodule important is that it could turn out to be malignant. The percentage of detection of malignancy among clinically and or radiologically detected thyroid nodules vary in available literature. The rate of malignancy, proven by various cytology or biopsy techniques, according to many studies done worldwide, ranges between 4% and 6.5% [5,6]. Ultrasonography remains the most widely employed modality in detection of nodules and sonographic features such as calcifications, margins, vascularity and echogenicity can determine the potential risk of malignancy of a thyroid nodule [7]. 

Nodules one centimetre or greater or sonographically suspect subcentimeter nodules require cytological investigation using fine-needle aspiration biopsy (FNAB). The main diagnostic tools a surgeon uses to decide the extent of thyroid surgery are with help of cytology and molecular biomarkers. Lately, ultrasonography-guided FNAB is appreciated as one of the key investigations in the primary evaluation of any thyroid nodule. Even the capacity of 18F-FDG positron emission tomography/computed tomography (PET/CT) to accurately distinguish between benign and malignant thyroid nodules was found to be moderate [8]. Hence there are no single modalities or standard criteria for easy diagnosis and interpretation of thyroid nodules. Though there are many thyroid-related studies, none of them show a significant impact on clinical practice. Hence we borrow the term triple assessment from the breast examination and implicate it in the evaluation of thyroid nodules as well.

## Materials and methods

All patients presenting with thyroid nodule laboratory assessment in a euthyroid state were studied over a time period of 18 months at SRM Medical College Hospital and Research Centre, Potheri. Seventy-five patients were included in this study. Patients who were assessed outside our hospital for cytopathology and ultrasonography reports were reassessed if they consented to the study. If the pathologists thought the smears were sufficient, slide reviews were accepted. The information needed for this study may not have been adequately reported from other ultrasounds, so all ultrasounds were performed again. A senior consultant conducted the clinical evaluation. Prior to doing the fine needle aspiration and cytology (FNAC), the designated radiologist performed the majority of the ultrasonograms. If the physicians believed it was necessary, ultrasound-guided FNACs were performed. Proforma was filled either in the outpatient area or following ward admission. Ultrasonogram is reported according to Thyroid Imaging Reporting and Data System- American College of Radiology (TIRADS - ACR) scoring and according to Bethesda criteria, the cytology was reported. The outcome of the histopathological analysis was used as the gold standard for diagnosis in this investigation.

Inclusion and exclusion criteria

All patients with thyroid swelling with a clinical euthyroid state (thyroid function test within normal limits) presenting to General Surgery OPD who consented were included.

Exclusion criteria were age <20 years or >70 years; ASA criteria III and above; previously undergone thyroid surgery; or patients not consenting to the study.

Statistical analysis

Cytology reporting was done as per the Bethesda criteria. The gold standard for diagnosis in this study is taken as the histopathology result. The study was conducted over the time period of 18 months beginning in February 2021 in SRM Medical College Hospital and Research Centre after ethical committee approval 2380/IEC/2021. The data were analysed using Statistical Package for the Social Sciences (SPSS), Version 28 (IBM Corp., Armonk, NY, USA), Python 3, Power BI and Microsoft Excel software (Microsoft, Redmond, WA, USA).

## Results

Table 1 depicts the occurrence of benign and malignant lesions across various age groups. One can clearly see that older patients (in the age category 50-70) had mostly malignant lesions (92%). In the younger age group (20-39), about only 11% were malignant and 77% had benign lesions. Overall, 41% had malignant lesions and 59% had benign lesions. The coefficient of correlation (R value) is 0.5, moderately positive.

**Table 1:** Comparison of age category across two groups R value is the coefficient of correlation HPE: histopathological examination

			HPE-1	HPE-0	
	Age Category		Malignant	benign	Total
	20-39	Count	11	36	47
		%	23%	77%	100%
	41-50	Count	9	7	16
		%	56%	44%	100%
	50- 70	Count	11	1	12
		%	92%	8%	100%
P value	0.00	Total	31	44	75
R value	0.5	%	41%	59%	100%

Table 2 depicts the occurrence of benign and malignant lesions across genders. While females had a 67% occurrence of benign lesions, 61% of males had malignant lesions as per histopathology reports. The R value is weakly negative.

**Table 2:** Comparison of sex across two groups

	Sex		Malignant	Benign	Total
	Male	Count	14	9	23
		%	61%	39%	100%
	Female	Count	17	35	52
		%	31%	67%	100%
P value	0.122	Total	34	41	75
R value	-0.3	%	55%	45%	100%

Table 3 depicts the correlation between a fixed swelling (clinically) and the occurrence of malignancy. It is noted that 73% of the fixed swellings turned out to be malignant. The R value is weakly positive.

**Table 3:** Comparison of fixed swelling/fixity across two groups

	Fixed Swelling/Fixity		Malignant	Benign	Total
	mobile swelling	Count	26	38	64
		%	41%	59%	100%
	fixed swelling	Count	8	3	11
		%	73%	27%	100%
P value	0.099378	Total	34	41	75
R value	0.2		45%	55%	100%

Table 4 indicates the correlation between extrathyroid extension and malignancy. About 86% of patients who had extrathyroidal extension ended up being found to have malignant lesions. Forty-one percent of patients who didn’t have any extrathyroidal extension also turned out to be having malignant lesions. The R value is weakly positive.

**Table 4:** Comparison of extrathyroid extension across two groups

	Extrathyroid Extension		Malignant	Benign	Total
	No Extension	Count	28	40	68
		%	41%	59%	100%
	Extension	Count	6	1	7
		%	86%	14%	100%
P value	0.063567	Total	34	41	75
R value	0.2		45%	55%	100%

However, the presence of pressure symptoms didn’t necessarily translate to being an indicator of malignancy. Only 38% of these patients turned out to be having malignant lesions. Also, among asymptomatic patients as well about 49% had malignant lesions, clearly indicating that the presence or absence of pressure symptoms doesn’t allow us to draw any conclusions towards the risk of malignancy. The R value has no association (Table 5).

**Table 5:** Comparison of pressure symptoms across two groups

	Pressure Symptoms		Malignant	Benign	Total
	Asymptomatic	Count	24	25	49
		%	49%	51%	100%
	Symptomatic	Count	10	16	26
		%	38%	62%	100%
P value	0.53059	Total	34	41	75
R value	0.0	%	45%	55%	100%

Table 6 depicts that clearly, punctate microcalcifications are an important marker of malignancy. About 97% of patients who had punctate microcalcifications turned out to have malignant lesions. The R value is strongly positive.

**Table 6:** Comparison of punctate microcalcification across two groups

	Punctate Mirocalcification		Malignant	Benign	Total
	No	Count	5	40	45
		%	11%	89%	100%
	Yes	Count	29	1	30
		%	97%	3%	100%
P value	0.00	Total	34	41	75
R value	0.8	%	45%	55%	100%

Hypoechogenicity on imaging also is an important marker of malignancy, with about 87% of patients who had hypoechogenicity having malignant lesions proven on histopathology. The R value is strongly positive (Table 7).

**Table 7:** Comparison of hypoechogenicity across two groups

	Hypoechogenicity		Malignant	Benign	Total
	No	Count	1	36	37
		%	3%	97%	100%
	Yes	Count	33	5	38
		%	87%	13%	100%
P value	0.00	Total	34	41	75
R value	0.8	%	45%	55%	100%

Sixty percent of patients who had an irregular mass or halo ended up having malignant lesions, while 68% of patients who didn’t have the above features had benign lesions, which indicates a correlation between irregular shape and malignancy. The R value is weakly positive (Table 8).

**Table 8:** Comparison of irregular mass/halo across two groups

	Irregular Mass/Halo		Malignant	Benign	Total
	No	Count	13	27	40
		%	33%	68%	100%
	Yes	Count	21	14	35
		%	60%	40%	100%
P value	0.03	Total	34	41	75
R value	0.2		45%	55%	100%

Table 9 indicates the relationship between dominant consistency and the occurrence of malignancy. All the patients who had solid lesions on imaging were proven to have malignant lesions. About 77% of patients who had cystic features ended up having benign lesions. Hence, it is a very significant marker.

**Table 9:** Comparison of dominant consistency across two groups

	Dominant consistency		Malignant	Benign	Total	Pvalue
Solid	No (0)	Count	10	41	51	0.00
		%	20%	80%	100%	
	Yes (1)	Count	24	0	24	
		%	100%	0%	100%	
Cystic	No (0)	Count	33	10	43	0.00
		%	77%	23%	100%	
	Yes (1)	Count	1	31	32	
		%	3%	97%	100%	
Mixed	No (0)	Count	24	32	56	0.6363
		%	43%	57%	100%	
	Yes (1)	Count	10	9	19	
		%	53%	47%	100%	
		Total	102	123	225	
		%	45%	55%	100%	

Intranodular vascularity, as per Table 10, is an important marker, as all patients who had intranodular vascularity on imaging were proven to have malignant lesions. The R value is strongly positive.

**Table 10:** Comparison of intranodular vascularity across two groups

	Intranodular Vascularity		Malignant	Benign	Total
	No	Count	5	41	46
		%	11%	89%	100%
	Yes	Count	29	0	29
		%	100%	0%	100%
P value	0.00	Total	34	41	75
R value	0.8	%	45%	55%	100%

Sixty percent of lesions that were taller than wide were proven to be malignant as per Table 11, thereby confirming its place as an important marker of malignancy in the thyroid. The R value is weakly positive.

**Table 11:** Comparison of taller than wide across two groups

	Taller than wide		Malignant	Benign	Total
	No	Count	16	30	46
		%	35%	65%	100%
	Yes	Count	18	11	29
		%	62%	38%	100%
P value	0.038126	Total	34	41	75
R value	0.3		45%	55%	100%

Lymph nodes are always an important indicator of malignancy. In our study, all patients with lymph nodes on clinical exam or imaging turned out to have a malignancy. However, the absence of lymph nodes doesn’t exclude malignancy, as seen by 25% of those not having lymph nodes also developing malignancy. The R value is moderately positive (Table 12).

**Table 12:** Comparison of lymph node across two groups HPE: histopathological examination

			HPE-1	HPE-0	
	Lymph Node		Malignant	Benign	Total
	No	Count	14	41	55
		%	25%	75%	100%
	Yes	Count	20	0	20
		%	100%	0%	100%
P value	4.44E-08	Total	34	41	75
R value	0.6		45%	55%	100%

**Figure 1:**
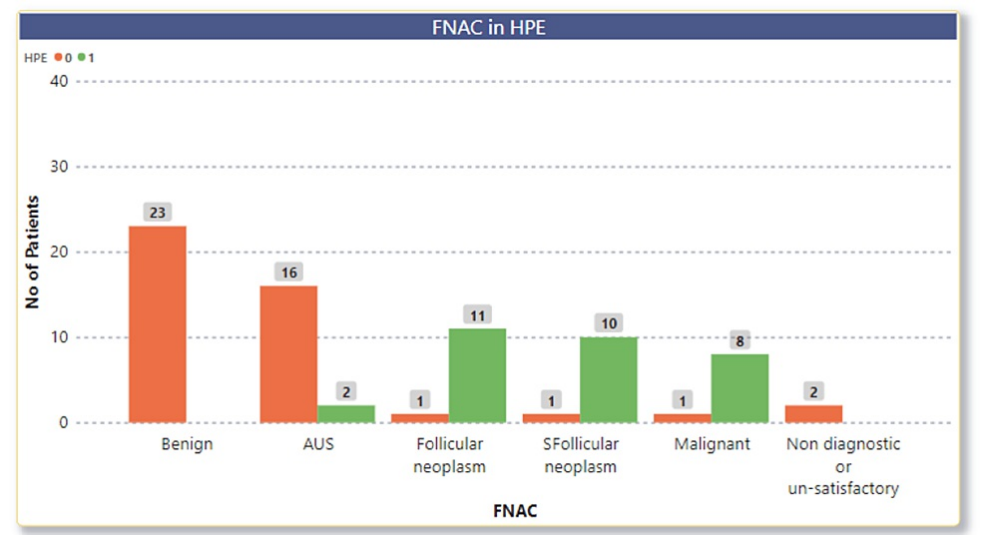
Fine needle aspiration and cytology (FNAC) in histopathological examination (HPE)

FNAC is an important diagnostic tool. It is made out that the reporting of FNAC matched the histopathological diagnosis in almost all categories (Table 13, Figure 1).

**Table 13:** Comparison of fine needle aspiration cytology (FNAC) across two groups HPE: histopathological examination

			HPE-1	HPE-0	
	FNAC		Malignant	benign	Total
	Non diagnostic or un‐ satisfactory	Count	0	2	2
		%	0%	100%	100%
	Benign	Count	0	23	23
		%	0%	100%	100%
	AUS	Count	2	16	18
		%	11%	89%	100%
	Follicular neoplasm	Count	11	1	12
		%	92%	8%	100%
	Suspicious for neoplasm	Count	10	1	11
		%	91%	9%	100%
	Malignant	Count	8	1	9
Pvalue	0.00	%	89%	11%	100%
		Total	32	43	75
		%	43%	57%	100%

Table 14 shows the R value of individual risk factors, also highlighting the overall importance of triple assessment in evaluating a thyroid nodule. Hypoechogenecity and intranodular vascularity are highly correlated as individual risk factors, and age and lymph node spread are moderately correlated as risk factors.

**Table 14:** Risk factor

Risk Factor	r Value
Age	0.5
Sex	-0.3
Fixity of Swelling	0.2
Extrathyroidal extension	0.2
Pressure Symptoms	0.0
Punctate microcalcifications	0.8
Hypoechogenecity	0.8
Irregular Mass/ Halo	0.2
Intranodular Vascularity	0.8
Taller than wide lesion	0.3
Lymph Node Spread	0.6

## Discussion

Malignancy was predominantly seen in males compared to females. Ill-defined margins, microcalcification, and taller-than-wide shape were ultra-sonography features showing correlations with malignancy. The study also states that overall rate of malignancy in patients undergoing surgery was 15.7% [9]. Ultrasonography and FNAB of the thyroid are popular, cost-effective, and easily applied diagnostic methods for most thyroid malignancies. Although inter- and intra-observer were seen when it comes to folicular adenoma/neoplasm [10,11]. Among the thyroid nodule diagnosed by FNA as indeterminate lesions found in 21% of cancers at histology [12]. It is to be noted that there is a study that demonstrates gender and age, but not ultrasound characteristics like microcalcifications, irregular margins, and marked hypoechogenicity, appear to influence the decision to perform surgery in atypia of undetermined significance/follicular lesion of undetermined significance (AUS/FLUS) patients [13].

As per other studies, ultrasonogram findings alone also do not provide reliable information. The single most feature with the best diagnostic performance was absence of elasticity. The most specific was the presence of central vascularization in ultrasonograms [14]. It was noted that taller than wide shape, absence of elasticity, presence of microcalcifications, and irregular margins were more frequently noted in lesions that turned out to be malignant post-test. But, none of these characteristics individually exhibited positive likelihood ratios (>10) or post-test probabilities in suspecting malignancy. So it was summarized that it was more likely a combination of these factors than any single individual element that may translate to a quantifiable risk for malignancy, but not many studies have addressed this issue [15].

Brito et al. demonstrated that being taller than wider lesions had the highest diagnostic odds ratio for judging the malignancy of thyroid nodules compared to other graphic ultrasound features [14]. Mai et al. concluded that even though there were no differences in gender, serum thyroid-stimulating hormone and free T4 (FT4) levels, thyroid auto-antibodies, thyroid dysfunction, and scintigraphic results between benign and malignant nodules. But groups with indeterminate cytology showing Bethesda System category IV and suspicious ultrasonogram features like border irregularities have high risk for malignancies. Among partially cystic lesions, those that show evidence of microcalcifications and eccentric configuration with an acute angle are deemed to be at higher risk. And features lie smooth free margin, peripheral vascularity, spongiform appearance or daughter cysts, and intranodular colloid crystals were signs of definite benignity [16].

However, in clinical practice, ultrasound features of thyroid nodules influence patient decisions, and final assessment of the nodules is based on various ultrasound features. Small (<2 cm) papillary thyroid carcinomas (PTCs) are responsible for nearly all of today's massive increase in thyroid cancer incidence. They are mostly seen in young but rarely fatal. PTCs in older adults are fatal. In order to overcome over-diagnosis, tumors that are <2 cm and present with clinical signs or symptoms must be biopsied and treated appropriately. There are really no binding guidelines regarding the management of indeterminate thyroid nodules, especially Bethesda 3 or 4.

Limitations

The constraints of time restricted the number of patients we could enroll in this study. Moreover, the sonological reports were from studies performed by different radiologists and pathologists. While we were able to establish a definitive role for triple assessment in the evaluation of thyroid nodule, further studies need to be done targeted at individual risk factors.

## Conclusions

Among clinical features, the size of the swelling and pressure symptoms did not correlate with a risk of being malignant. Nodules that were hard in consistency or exhibited fixity had a correlation with final diagnosis of malignancy The widespread use of ultrasound imaging to guide clinical decision-making is validated. Sonographic features with an individual correlation with malignancy include punctate microcalcifications, hypoechogenicity, intranodular vascularity, solid consistency and significant lymph nodes. Hypoechogenicity and intranodular vascularity are highly correlated as individual risk factors whereas age and lymph node spread are moderately correlated as individual risk factors. Overall, there is definitely a correlation in the role of triple assessment in the diagnosis of thyroid nodule and guiding its management. However, further studies are required to target individual risk factors more.

To conclude, ultrasound features can be a strong predictor for malignancy, and there is a definitive role for triple assessment (clinical examination, imaging and cytology) in the evaluation of thyroid nodules. 
